# Evaluating the impact of engaging older adults and service providers as research partners in the co-design of a community mobility-promoting program: a mixed methods developmental evaluation study

**DOI:** 10.1186/s40900-023-00523-5

**Published:** 2023-12-08

**Authors:** Maggie MacNeil, Julia Abelson, Caroline Moore, Shazelle Lindsay, Janet Adams, Aref Alshaikhahmed, Kamal Jain, Penelope Petrie, Rebecca Ganann

**Affiliations:** 1 School of Nursing, McMaster University, Hamilton, ON Canada; 2 Department of Health Research Methods, Evidence and Impact, McMaster University, Hamilton, ON Canada; 3 EMBOLDEN Strategic Guiding Council, Hamilton, ON Canada

**Keywords:** Patient engagement, Patient-oriented research, Patient and public involvement, Evaluation, Older adults, Co-design, Stakeholder engagement, Community-based, Citizen partners

## Abstract

**Background:**

Increasingly researchers are partnering with citizens and communities in research; less is known about research impacts of this engagement. EMBOLDEN is an evidence-informed, mobility-promoting intervention for older adults co-designed by a 26-person Strategic Guiding Council (SGC) of health/social service providers and older adult citizens. This study evaluated research partners’ perceptions of engagement strategies, the engagement context, strengths, areas for improvement, as well as the impacts of the guiding council on older-adult identified priority areas.

**Methods:**

This study was guided by developmental evaluation, working in partnership with four older adult SGC members who helped to set evaluation priorities, decide methods, and adapt patient-centred evaluation tools. Data sources included a questionnaire, focus groups and document analysis of meeting notes from 16 SGC meetings that took place between December 2019 and February 2022. A thematic approach to analysis guided the coding of focus group transcripts and SGC meeting notes. Convergent mixed methods guided the integration and presentation of qualitative and quantitative data sources in a joint display of evaluation results.

**Results:**

Of 26 SGC members, nine completed the evaluation squestionnaire, and five participated in focus groups. Around two thirds of the SGC commonly attended each meeting. EMBOLDEN’s SGC was structured to include a diverse group (across gender, ethnicity and discipline) of older adults and service providers, which was perceived as a strength. Engagement processes were perceived as inclusive and well-facilitated, which stimulated discussion at meetings. Advantages and disadvantages of engaging with the SGC virtually, as compared to in-person (as was the case for the first 3 SGC meetings) were also discussed. Impacts of the SGC were identified across preparatory, execution phase and translational stages of research. Impacts of SGC involvement on members were also described.

**Conclusion:**

Older adult research partners played an important role designing, implementing, and evaluating co-design approaches in this study. Older adults and service providers can make important contributions to the design, delivery and sharing results of health research through their lived expertise and connections to community. This project contributes to the growing field of citizen and community engagement in research by offering a participatory approach to engagement evaluation that considers diversity, satisfaction, and impact.

**Supplementary Information:**

The online version contains supplementary material available at 10.1186/s40900-023-00523-5.

## Abstract (plain language summary)

It is becoming common for researchers to ask patients and caregivers to become involved with their studies as partners. By partnering with people who have first-hand experience with a condition, researchers hope their studies will be more relevant, run efficiently and have more impact than studies designed by researchers alone. Over the last twenty years, there has been a significant increase in the number of published papers that involve patients and caregivers, but few papers mention what changed because of this involvement. Researchers need to know the best ways to involve patients and caregivers and the impact of doing so.

This study aimed to find out how a council of older adults (OAs) and service providers (SPs) impacted a study about collaboratively designing and testing a mobility intervention. This study aimed to find out how council members felt about their involvement in this study, by asking them what went well and what could have been improved.

This study used a questionnaire, focus groups and analyzed council meeting notes to inform this study. Four OA council members helped design the study, decide research methods, and adapt wording of questions (focus groups, questionnaire). Between 19% and 35% of council members completed evaluation activities. They felt the study strengths were the diversity of the council, and a welcoming environment for discussion. The council impacted how the study was designed, carried out and how results were shared. Results from this study can improve patient partnership engagement, experiences, and impacts in future studies.

**Supplementary Information:**

The online version contains supplementary material available at 10.1186/s40900-023-00523-5.

## Background

Patient-oriented approaches to research continue to be prioritized locally, nationally, and internationally [1]. Canada’s Strategy for Patient Oriented Research (SPOR) envisions patients as active partners in health research to improve health outcomes and enhance the health care system [2]. The Canadian Institutes of Health Research (CIHR) defines patient engagement as “meaningful and active collaboration in governance, priority setting, conducting research and knowledge translation” by ‘patients’, a term that is broadly used to include individuals that have had a direct personal lived experience with a health issue, as well as caregivers, including family and friends [2]. The term ‘patient and public involvement’ is the widely used term for this practice in Europe and the UK [3]. Both terms reflect the idea of doing health research with people with relevant lived health and healthcare experiences, as opposed to doing research for them.

Patient engagement has been recognized as a relevant, credible, and egalitarian approach to health research, and has become structurally integrated into the Canadian health research enterprise [4–6]. Even so, details about the impacts of engaged approaches on research are reported inconsistently in the rapidly expanding patient engagement literature [7, 8]. Rigorous evaluation of engaged approaches can support comparison and capacity building among researchers to ensure that limited resources are allocated to the most impactful proposals [7, 9]. Consensus has formed around the need to strengthen the evidence base for patient-engaged research, though there is less agreement about how best to accomplish this outcome [7, 10, 11]. Tools such as the Patient and Public Engagement Evaluation Tool (PPEET) [12]; the Ways of Engaging-Engagement Activity Tool (WE-ENACT) [13] and the Patient Engagement in Research Scale (PEIRS) framework [14] are common approaches to evaluating impacts of patient engagement. Previous research has assessed the PPEET and the Patient-Centered Outcomes Research Institute (PCORI) Engagement Activity Inventory (which includes the WE-ENACT tool) on their scientific rigor, patient and public perspective, comprehensiveness, and usability [7, 15]. The PPEET received 80% or higher on all criteria, and the PCORI Engagement Activity Inventory scored 80% or higher on comprehensiveness and usability, with room for improvement in scientific rigor (20%) and level of patient and public engagement in design and reporting (60%) [7, 15]. In a subsequent analysis, the PEIRS framework received 80% or higher in scientific rigor and usability, with slightly lower scores (60%) in its incorporation of patient and public perspective and comprehensiveness [16]. In addition to the work to appraise existing tools, in Canada, patient-oriented evaluation tools have emerged that support the impact evaluation of engagement on specific sectors, including the health system [17] and on health research outcomes [18].

### Challenges associated with evaluating engaged approaches to health research

Previous studies have shown that patient involvement is occurring at various points of the research cycle and can lead to improved health services, however, less detail is available on other impacts of engagement i.e., the cost and impacts of engaged approaches [19, 20]. Patient engagement studies that do report on engagement impacts, often use anecdotal reflections of researchers [21] or tools that were developed without input from patients [7]. Newer frameworks about patient engagement in health research are reportedly not well-connected to more established theories of community-based participatory research, a longer-standing tradition based on the full, equal partnership of community members in all aspects of research [22, 23]. The silos of engagement theory and inconsistent terminology that have been identified between health and social science [4] can be confusing for researchers new to this field [22]. For example, one review found over 15 conceptual terms currently in use to describe patient engagement [24]. Carroll and colleagues [25] suggest that this complexity in terminology makes it hard to understand what outcomes should be measured and what methods should be used to generate results when evaluating patient-engaged approaches to health research. This fragmented evidence base of impact evaluation in patient engagement makes it challenging to understand what engagement approaches work best for what type of patient partners and why; in turn, this makes it challenging for novice researchers to determine whether they are engaging patients in a tokenistic or authentic way [8, 25]. To address variability and gaps, and to promote rigorous reporting in the field, Guidance for Reporting Involvement of Patient Partners (GRIPP2) has been developed to standardize reporting about involvement and impact of patient partners in published research [8]. Standard ways of reporting engagement can increase transparency about: in/effective approaches to engagement and how to best measure impact while promoting consistent terminology and bridging theoretical traditions—moving the growing field of engagement researchers forward. Additional file 1 contains reporting about the involvement of patient partners in this study. 

### Evaluating engaged approaches with older adult research partners

As patient-engaged approaches become more common, so too does the practice of involving older adults as research partners [26]. It is not uncommon for researchers to comment on the outcome of engaging older adults. For example, Evans and colleagues report that older adult research partners revised study procedures, recruited/interviewed participants, and led presentations to share research findings [27]; Jansen and colleagues describe a co-designed framework to facilitate older adults’ involvement in national policymaking [28]; and Kelly and colleagues report improved engagement processes in a patient advisory committee as a result of older adults’ contributions [29]. More recently, multi-method approaches to evaluating (as opposed to describing or reflecting on) impacts of engaging older adults in research have been reported [18, 30]. These include the use of a rating scale, interviews and focus groups to measure older adults' involvement in a multi-site dementia research study [31], and a participatory mixed-methods approach to co-develop a tool to screen for medication use problems [32]. Despite the growth of engaged approaches to research with older adults, and the trend towards measuring impacts of older people’s engagement (as opposed to describing them), there is a dearth of literature on partnered approaches to evaluating the impact of older adults’ engagement in research. Developmental evaluation is an established form of participatory evaluation [33, 34] that is well-suited to capture the complexity of co-designed research, yet underutilized in the measurement of engagement impacts [35, 36]. In partnership with older adults, this study aimed to evaluate the co-design process used with a Strategic Guiding Council (SGC) who were engaged in designing a mobility-promoting community-based program for older adults. Specifically, we aimed to understand how research partners perceived engagement strategies, the engagement context, what worked well, and areas for improvement, as well as the impacts of the guiding council on older-adult identified priority areas. This study aims to address a gap in rigorous, participatory approaches to evaluating the impact of engaging older adults as research partners.

## Methods

### Research context—the EMBOLDEN study (Enhancing physical and community mobility in older adults with health inequities using community co-design of a complex intervention incorporating exercise, nutrition, social participation, and system navigation)

The evaluation study was carried out within the context of a larger research project (the EMBOLDEN study) which aimed to co-design a mobility program for older adults aged 55 + years who live in neighborhoods identified as having high rates of health inequity in Hamilton, Ontario, Canada [37]. An Experience-Based Co-design (EBCD) approach was used, which aims to improve healthcare services by having patients/citizens, caregivers and health/social service providers reflect on their experiences and work to identify priorities for improvement and processes to enhance health services [38, 39]. The EMBOLDEN research team partnered with a SGC composed of 8 older adults and 18 community health and social service providers, to co-design a program to promote community and physical mobility, healthy eating, social participation, and system navigation of older adults living in neighborhoods facing health inequities. The impacts and scalability of the EMBOLDEN program will be measured in a pragmatic randomized controlled trial (RCT) in Hamilton and Toronto, ON [40].

Some SGC members were confirmed and named in the research proposal and helped with snowball sampling to recruit a broad range of community groups, including city housing, public health, community health centres, libraries, seniors’ advisory councils, and neighbourhood associations to include in the SGC. A targeted sampling approach, with referrals from community partner organizations was used to ensure diverse older adults were recruited to the SGC. To complement snowball sampling, some consenting older adults who had been participants in an EMBOLDEN-affiliated qualitative study (about their experiences with local community programming) were invited to join EMBOLDEN’s SGC. Older adult citizen partners were reimbursed for any travel expenses and received an honorarium of $25 per hour for their participation in SGC meetings and for the support provided to this evaluation sub-study.

### Approach

In developmental evaluation (DE), the evaluator and ‘social innovators’ develop the evaluation together and the evaluation becomes part of the program [41, 42]. DE’s emphasis on working with program users throughout the evaluation process is to ensure the utility and use of evaluation results [41–43]. This process aligns well with the role of the SGC within the EMBOLDEN program more broadly: to act as research partners in the collaborative co-design, testing, and planning for future uses of the EMBOLDEN program. With a DE approach, special attention is paid to interrelationships and the complex context in which the program and the evaluation are embedded, helping to account for the complexity of this co-design effort (16 meetings between December 2019 and February 2022) [35, 42]. EMBOLDEN’s co-design process with its SGC transitioned from initial in-person meetings to virtual meetings due to public health restrictions related to the COVID-19 pandemic. Three of 18 meetings covered in this evaluation occurred in-person, followed by a 4-month pause on SGC activities based on university and research ethics board (REB) pandemic guidance, and continued virtually for the remaining 15 meetings. Table 1 represents a timeline and topic summary of SGC activities across the evaluation period, more information about the co-design process can be found in Ganann and colleagues [40]. It should be noted that only some research team members were part of regular SGC meetings, to ensure meetings focused on older adult and service provider perspectives. Table 1 also contains counts of SGC members and researchers present at each meeting.

**Table 1:** EMBOLDEN SGC timeline

Date	Main topics covered, number of SGC members present, number of research team present	SGC	Research team
Dec. 2019	Launch Event: expectations for working together; understanding target neighborhoods; identifying priority features; understanding barriers to overcome	18	23
Jan. 2020	What we learned at the Launch Event (core program features, timeline, processes), EMBOLDEN’s core outcome measure	15	4
Feb. 2020	RCT outcome measures, system navigation	15	4
*March–August 2020: Research pause due to COVID-19 pandemic*			
Aug. 2020	COVID-19 impact on current city programming/vision of EMBOLDEN going forward	17	7
Sept. 2020	Overview of social participation: creating an inclusive, anti-racist environment; social prescription; outcome measures	12	3
Oct. 2020	Overview of physical activity (types, supporting evidence)	16	6
Nov. 2020	Overview of healthy eating (nutrition risk, food insecurity, resilient healthy eating behaviors); supporting evidence; barriers/facilitators/supports, exemplary healthy eating programs	19	6
Jan. 2021	Framing/building interest for program; length/frequency/duration/staffing of program	15	6
Feb. 2021	Impact of COVID-19 on community, organizations, reopening, mode of delivery; fixed vs. flexible program components	14	4
Apr. 2021	Identifying pilot neighborhood; active vs. passive recruitment, supporting evidence; reaching target population; key messages; role for SGC; recruiting for engagement evaluation study	19	7
May 2021	Planning physical activity: interpreting supporting evidence; describing physical activity; interventionist; who/how/when system navigation support occurs; exemplary programs	12	7
Jun. 2021	Planning healthy eating: interpreting supporting evidence; experiential virtual healthy eating, foods/topics of interest, cultural elements; outcomes package; defining research concepts (e.g., RCT, REB)	14	5
Jul. 2021	Reviewing ethics materials (e.g., program satisfaction surveys, post-program program evaluation questions, recruitment materials); planning social component (e.g., fostering interactions online vs. in-person)	14	6
Sept. 2021	Operationalizing aspects of exemplary programs for use in EMBOLDEN, presenting EMBOLDEN logic model, interpreting qualitative study results (e.g., emotional touchpoints), training plan for intervention team	14	7
Oct. 2021	Focus groups 1 and 2, engagement evaluation questionnaire shared with SGC members		
Nov. 2021	Community Advisory Board recruitment; trial recruitment timeline; peer facilitation; training materials for intervention team	16	6
Feb. 2022	EMBOLDEN communications plan, discussion/interpretation of engagement evaluation results (questionnaire and focus group). End of evaluation period	12	6
Jun. 2022	Discussion/interpretation of engagement evaluation results re: impact (document analysis)		

The DE approach also accommodates the use of multiple data sources allowing researchers to present both quantitative and qualitative research approaches to capture data on priorities identified by research partners [43].

### Designing the evaluation

In May 2021, SGC members were invited to attend an additional meeting with a sub-group of the research team to help design the evaluation of the co-design process. Of the 26-person guiding council, four older adults (two men and two women) expressed interest and availability to partner with this sub-group of the research team to design this sub-project. One researcher led the session; another took notes. Meetings lasted 60 min and were conducted using Zoom videoconferencing software [44]. Ethics approval for this project was provided by the Hamilton Integrated Research Ethics Board (project #7668).

Prior to the first meeting, the older adult research partners were given a 1-page description of the background and rationale for the DE project, a description of what to anticipate during the session, who would be involved, and discussion questions to consider (See Appendix A). The group of research partners collectively identified that the following values and outcomes should be evaluated: diversity, respect, satisfaction, and SGC contributions to the various stages of research and impacts. During the meeting, a list of strategies to capture data to inform the evaluation was displayed (individual interviews, observation, focus group, questionnaire, process metrics (counts of time, attendance, meetings) and older adults identified a questionnaire and focus groups as suitable methods to capture data about the values and outcomes they had identified earlier in the meeting. They felt that focus groups would be a useful source of detailed feedback, but an anonymous questionnaire that was quick to administer and could create a safe means environment to share any negative feedback. The group preferred that the duration of the focus groups not exceed 45 min in length. Researchers proposed that SGC meeting notes could be analyzed to trace impacts of the SGC across the various meetings; the older adult partners agreed this would be an efficient approach to enhance transparency with respect to how SGC input contributed to EMBOLDEN study decision-making.

Prior to the second meeting, draft data collection tools were shared with the older adult research partners. These included focus group prompts adapted from the WE-ENACT tool [13] and a questionnaire adapted from the PPEET tool [12]. Partners also received a rubric showing which questions on each tool captured the key metrics they had previously identified (diversity, respect, satisfaction, and the contributions/impacts of the SGC across all stages of research) (Table 2). Recognizing there is no ‘one size fits all’ way to evaluate engaged approaches to health research [9, 18, 45], older adults tailored (by removing, re-wording and re-ordering questionnaire questions and focus group prompts) measurement tools. For example, research partners suggested changing the order of PPEET questions (to discuss the adequacy of information before discussing supports for their role), reworded questions to reflect the present tense (as engagement was still ongoing) and added language to the demographic question about highest level of school completion to change ‘no schooling’ to ‘no formal schooling’. Their suggestions to WE-ENACT tool to included asking about how prepared SGC members felt to contribute, supports members would have liked to have received, and if/how involvement has changed their lives, before asking about the impacts they feel they have had on the project. (See Appendices B and C for more detail on evaluation tools).

**Table 2:** Evaluation Domain and data sources

Domain	Data source and item	Prioritized by
Diversity (culture/ethnic; organizations, gender)	Questionnaire (Q3, Q4, Q5, Q13, Q14) Meeting notes	SGC older adults
Respect	Questionnaire (Q11, Q12)	
Satisfaction	Questionnaire (Q21, Q22)	
SGC involvement across stages of research; impact of SGC on program	Focus Group Interview Guide (Q5) Questionnaire (Q17, Q18, Q19) Meeting notes	
Strengths/indications of success	Focus Group Interview Guide (Q8) Questionnaire (Q23)	Research question/DE
Challenges/areas for improvement	Focus Group Interview Guide (Q7) Questionnaire (Q24)	

### Conducting the evaluation

A mixed methods approach to DE integrating a questionnaire, two focus groups, and document analysis of meeting minutes from 16 SGC meetings was used. Aligned with a convergent mixed methods approach, the qualitative and quantitative data were collected in parallel to iteratively inform the final evaluation results [46]. The reporting of findings is aligned with Tong and colleagues’ Consolidated Criteria for Reporting Qualitative Research (COREQ) [47]. Two systematic reviews created and advanced a taxonomy to characterize patient involvement in research, identifying when, engagement occurs during the research cycle, which were helpful in the conceptualization of impacts across the research cycle [48, 49].

In October 2021, an email invitation was sent to all SGC members to invite them to complete a questionnaire and to participate in a focus group to explore their perceptions of the process of engagement in EMBOLDEN’s SGC and engagement impacts. The lead of this DE sub-project was known to SGC members and was given time during virtual SGC meetings to promote participation in the evaluation project (questionnaire and focus group). Questionnaire data were collected using LimeSurvey, and analyzed using descriptive statistics [50]. Focus groups were conducted using Zoom software [44], audio-recorded and transcribed verbatim. The lead author facilitated the focus groups, while another researcher took notes. Focus group transcripts, short answer questionnaire data and meeting minutes were analyzed with NVivo 12 [51] using thematic analysis, to identify, analyze, and reports patterns or themes within data [52]. This process was completed by the study lead and checked by a co-author. The original coding tree included deductive codes corresponding with evaluation domains in Table 2. The coding team inductively derived sub-themes labels, and definitions; any discrepancies were resolved by debriefing meetings occurring throughout the coding process. Themes were reviewed by the investigator team to ensure they were supported by data. Preliminary results from the questionnaire, focus groups, and document analysis of meeting minutes were presented to the SGC in two meetings, (February and June 2022) to seek their guidance in interpreting these findings. Subsequently, authors developed a joint display, integrating results from qualitative (short answer questionnaire data, focus group transcripts, meeting notes analysis) with quantitative (questionnaire results) [46, 53]. Table 2 supported integration by identifying which prompts/questions from which data source informed different domains of the evaluation [43, 46, 53]. The joint display (Table 3) allows for more complete inferences about the evaluation domains, which integrate data from different sources to explain and triangulate the impacts, strengths, limitations and perceptions of engagement regarding partner-identified priorities [53].

**Table 3:** Characteristics of evaluation questionnaire participants (n = 9)

Age range	Older adults	65–77 years
	Service providers	31–58 years
Gender	Female	66%
	Male	33%
Education	Completed college	22%
	Completed university	22%
	Completed post/graduate/professional degree	56%
Ethnicity	Arab/West Asian, South Asian, White/Caucasian	11% 11% 78%
Identify as	LGBTQ	11%
	Persons with disabilities	22%
	Recent immigrant	11%
	Visible minority	11%

## Results

The evaluation results will be presented as they relate to the structure of the SGC, SGC processes, and impacts of the SGC, drawing on all data sources. Between 19% and 35% of the SGC participated in evaluation activities, compared to their engagement with regular SGC meetings, where, as Table 1 shows, attendance ranged from 46% to 73%. A convenience sample of five SGC members (4 older adults; 1 health/social service provider) participated in two focus groups while nine SGC members (5 community service providers, 4 older adults) completed the engagement evaluation questionnaire. Table 3 includes demographic data about evaluation questionnaire participants. Sociodemographic data were not collected from focus group participants, or the broader SGC membership.

### Structure of the SGC

As seen in Table 4, most questionnaire respondents agreed or strongly agreed with evaluation statements about respect, satisfaction, and diversity. Table 5 demonstrates that older adult respondents felt slightly stronger in their agreement with evaluation prompts than service provider respondents. The linkages between diversity, satisfaction and respect are detailed in the following excerpt:

I have not felt that there has been a hierarchy among the participants. Everybody was given the same respect in terms of speaking or listening. Moreover, everybody was encouraged to provide his/her points of view. I was happy and satisfied by my participation because I was working with a team who enjoyed a high level of trust and sincerity to achieving the objectives of the study. We listened to each other with respect.

- Older adult questionnaire respondent

**Table 4:** Engagement evaluation questionnaire results (n = 9)

Domain	Evaluation statement	Level of agreement				
		Strongly disagree (%)	Disagree	Neither agree nor disagree	Agree (%)	Strongly agree (%)
Respect	I am able to express my views freely	11	0	0	22	67
	I feel that my views are heard	11	0	0	33	56
Diversity	A wide range of views on the topics discussed are shared	11	0	0	33	56
	Members of EMBOLDEN’s SGC represented a broad range of perspectives on the topic	11	0	0	33	56
Satisfaction	I am satisfied with my engagement in the SGC	11	0	0	33	56
	Participating in the SGC is a good use of my time	11	0	11%	33	45
Impact	I am confident the input provided to the SGC is heard	11	0	0	22	67
	I think my input will make a difference to the work of the EMBOLDEN research team	11	0	11%	44	33

**Table 5:** Evaluation results table

Values		
Domain	Data source and item	Result, representative excerpt
Diversity	Q3: what is your gender?	Older adults: 50% male, Service providers: 20% male
	Q4: are you a member of any of the following groups: (LGBTQ, recent immigrant, persons with disabilities, visible minority)?	Older adults: 75% identified with ≥ 1 equity-seeking groups Service providers: 40% identified with ≥ 1 equity-seeking groups
	Q5: Which racial or ethnic group do you belong to?	Older adults: 50% Caucasian Service providers: 100% Caucasian
	Q13: A wide range of views on the topic are shared	Older adults: 100% agreement Service providers: 80% agreement
	Q14: The individuals …represented a broad range of perspectives on the topic?	Older adults: 100% agreement Service providers: 80% agreement
	M6	“*What would you tell friends, family and colleagues about EMBOLDEN? Based on what you know now? Cross-sectoral, academic rigor, base of expertise, and diverse voices being heard, and it is well-funded.”*—6^th^ SGC Meeting Notes
Respect	Q11: I am able to express my views freely	Older adults: 100% agreement Service providers: 80% agreement
	Q12: I feel that my views are heard	Older adults: 100% agreement Service providers: 80% agreement
Satisfaction	Q21: I am satisfied with my engagement	Older adults: 100% agreement Service providers: 80% agreement
	Q22: Participating in the SGC is a good use of my time	Older adults: 100% agreement Service providers: 75% agreement
Impact	Q17: I am confident the input provided by the SGC is heard by the research team	Older adults: 100% agreement Service providers: 80% agreement
	Q18: I think the input provided will make a different to the work of the research team	Older adults: 100% agreement Service providers: 75% agreement
	Q19: In your role, what influence have you had to date? (short answer)	Older adult excerpt: “*I have seen and heard my view, as well as others, come back through future meetings.”* Service provider excerpt: *“Sharing clinical experience with programming for Seniors.”*
	FG5: In what ways do you feel your engagement in the SGC influenced the EMBOLDEN program?	Older adult excerpt: *‘I think some people have offered to help find older adults to participate because they either live in one of the areas that the study’s gonna take place in or they know people who live there so … you know and probably without this SGC that that would possibly not have happened. the SGC has done a lot of this’* Service Provider excerpt: *“I think sharing the outcomes of the research to know that you contributed to something… like in everything we do with the research, when we’re actually part of the implementation and work we get named in the publications, so I think sharing the outcomes and seeing where it landed”*

Q: Questionnaire, FG: Focus group, M : Meeting, agreement: Agree or strongly agree

Across the questionnaire results and focus group findings, the diversity achieved in the structure of EMBOLDEN’s SGC was seen as a notable strength of the study. Table 5 demonstrates that older adult questionnaire respondents were more diverse across gender, ethnicity, and self-identification with equity-seeking groups than service providers who responded.

### Engagement processes: indications of success

#### Engagement style

Participants spoke about the style of engagement as a strength of the project. Specifically, they expressed that the atmosphere within the EMBOLDEN SGC encouraged discussion, and that they felt welcome and included within the project. SGC experiences were described in the following quote:

Right from the very beginning I felt welcome. You know that the team that were putting this project together wanted me and wanted everyone else in the room to be there. You know it wasn’t like … you know we have to do this for show because our granter expects us … you know, the place that’s giving us the money, expects us to engage the community somehow, so we have to do this. No, I mean I felt right from the beginning that they wanted…this group in the room talking about the project. – Older adult 3, focus group 2.

A second attribute of the researchers’ engagement style that was mentioned as a strength of the project, was the reporting style. Focus group data and meeting notes confirmed SGC members’ emphasis on the importance of circling back to research partners on how their input was used, or as one service provider put it: “*sharing the outcomes of the research to know that you contributed to something”* -Service Provider 1, focus group 2.

#### Organization

Across multiple data sources, organization was identified as a strength of EMBOLDEN’s approach to co-design. For evaluation participants, organization presented as being responsive and communicating clearly. One service provider questionnaire response exemplified much of the feedback on the co-design’s organization: “*Virtual meetings are well organized and conducted. Excellent coordination and support to members is provided*”. A service provider noted: *I think it goes back to being really organized so I think that makes a difference that … you know stuff’s not coming in pockets. It was always an agenda, here’s minutes… really quick to respond*”—Service Provider 1, focus group 2. Others conveyed appreciation for EMBOLDEN researchers’ ability to simplify concepts and avoid jargon.

#### Advantages of virtual engagement

Participants identified in both focus groups and SGC meetings that virtual meetings were convenient to attend as partners did not have to look for parking, navigate to an unfamiliar meeting space, and saw virtual communication as inevitable to allow collaborations to continue during the COVID-19 pandemic. Smaller break out groups were seen as key elements indicating success. Across data sources, the use of virtual break out rooms during SGC meetings was unanimously identified as a strength of EMBOLDEN’s approach to co-design, with an older adult noting:

I think the general meetings were very good for you guys to tell us what’s going on, but for us to tell you or give you some thoughts or suggestions, I believe practically all of them came from the breakout groups – Older adult 2, focus group 1.

Due to the convenience of virtual meetings identified by SGC members, they continued online even after pandemic restrictions were lifted.

### Engagement processes: opportunities to improve

Focus group discussions centred around the strengths of EMBOLDEN’s SGC. Participants initially did not to have suggestions on how to improve EMBOLDEN’s engagement approaches, however, when prompted to further to identify advice they would give to future researchers co-designing research, they offered strategies to enhance meeting logistics and virtual engagement.

#### Meeting logistics

Some participants identified the importance of planning for parking (for in-person meetings), allowing for a break during long meetings, and the value of sharing meeting materials in advance. When preliminary evaluation findings were shared with the SGC, some members commented on low participation rates for the evaluation and suggested protecting time during scheduled meetings for evaluation activities to increase participation rates rather than as a separate and additional activity.

#### Challenges of virtual engagement

Through focus groups and SGC meeting notes, the tiring nature of virtual meetings was identified, noting that it was harder to pay attention without all the stimuli associated with meeting in-person. Participants spoke about how the ability to turn off one’s zoom camera can enable distractions and gives the impression of being *“available, but not available”*—Older adult 1, focus group 1. Participants expressed that multiple virtual meetings in a single day can be challenging and empathized with colleagues who appeared to have multiple virtual meetings scheduled back-to-back: “*you know the joy of digital is that we can meet and the pain of it is that because we can meet and it’s online, they can just be one after the other”*—Older adult 3, focus group 2.

### Impact of SGC across stages of research

As seen in Table 4, questionnaire respondents showed some of the strongest agreement of any question that their input was heard by EMBOLDEN researchers, and most respondents agreed that SGC input makes a difference to the research team. The following response offers a good summary of SGC impact: ‘*I have seen and heard my view, as well as others, come back through future meetings’*- older adult questionnaire response.

We drew on a previous taxonomy to conceptualize the impacts of the SGC across preparatory, execution, and translational stages of research [48, 49]. Table 6 summarizes the SGC impacts across research stages that were documented or shared.

**Table 6:** Results summary

Evaluation domains					
Strengths and opportunities	Values	Impacts			
		Phase of the research project			On participants
*Strengths*	*Diversity*	Preparatory phase	Execution phase	Translational phase	Knowing and doing more physical activity
Engagement: - Atmosphere encourages discussion - Felt welcome, included, heard	SGC members: - Are age, gender diverse - Identify as members of equity-seeking groups - Agree that wide range of views/perspectives are shared	Adding diverse voices	Interpreting evidence to identify priority features	Co-authored knowledge products: - Infographics - Research briefs - Study website - Conference presentations - Peer-reviewed publications	Sharing knowledge and experience
Organization: - Supportive, responsive - Clear communication Advantages of virtual engagement: - Convenience - Break out groups	Older adult SGC members are ethnically diverse	Choosing meaningful language	Helping researchers understand community		Desire to continue research partnerships
*Opportunities*	*Respect*		Refining outcomes		
Challenges of virtual engagement: - Screen fatigue - Meet in-person first	The majority of SGC members agree that they: - Can express their views - Feel their views are heard		Transitioning to virtual pilot		
Meeting logistics	*Satisfaction* The majority of SGC members: - Are satisfied with the SGC - Think it is a good use of their time				

#### Preparatory impacts

Beginning with the first meeting to launch EMBOLDEN’s SGC in December 2019, meeting minutes reported the emphasis on centring diverse older adults from EMBOLDEN’s target neighborhoods, in addition to the older adults who were already involved with EMBOLDEN. In response to this, EMBOLDEN researchers worked with SGC partner organizations to recruit additional older adults representing broader experiences relevant to EMBOLDEN (e.g., diversity in gender, ethnicity, neighbourhood). A second impact of the SGC, which occurred primarily during the early preparatory stages of the project, was their contributions to helping researchers understand the community’s assets and gaps.

Since the inception of the SGC, service providers and older adults helped to contextualize demographic information and inventories of programs/services in each of EMBOLDEN’s target neighborhoods. For example, SGC members helped identify programs that were missed by EMBOLDEN’s foundational environmental scanning and provided detailed information about the availability and, in some instances, quality of neighborhood-level programs and services. The SGC advised on local impacts of the COVID-19 pandemic at a time when these services underwent rapid shifts in delivery models and service availability, and little information was publicly available. For example, meeting notes report a significant reduction in accessible transit use and the radical increase in uptake of mental health programing. One older adult focus group participant spoke to the broad nature of SGC efforts to help researchers understand the community: *“we are kinda offering you suggestions in the spirit of, I don’t know if guidance is the right word…we may be of help, to some extent, to suggest ways for you to keep on track”*– Older adult 2, focus group 1.

The SGC played an integral role in choosing meaningful and appropriate language to describe and promote the EMBOLDEN program. For example, while EMBOLDEN funders identified nutrition as a priority area for the program to focus on, in working with the SGC to operationalize ‘nutrition’ it became apparent that this term was too narrow. SGC members pointed out that ‘nutrition' presumes access to healthy food, which is not the case for many older adults who may be limited by cost of food, ability to transport healthy food, availability of healthy food, or ability to prepare healthy food. The ‘nutrition’ priority area was then renamed ‘healthy eating’ to reflect both access to and consumption of healthy food.

#### Impacts on the execution of research

The main role of the SGC was to identify the priority features of the EMBOLDEN intervention. This was achieved through considering qualitative and quantitative research evidence (identified through foundational projects) and operationalizing these findings to the context of EMBOLDEN’s target neighborhoods. Though the detailed activities of the co-design process are described elsewhere [40], analysis of meeting minutes helped to trace specific contributions of SGC related to each of EMBOLDEN’s core program components: healthy eating, physical activity, socialization, and system navigation. SGC comments around physical activity emphasized the importance of prioritizing specific exercises with their real-world functional value to older adults, for example, focusing on maintaining strength to support daily functioning, such as carrying groceries or kitchen activities. In an SGC meeting, members described exemplary programs where *‘you could see older people spring up friendships’(*6^th^ SGC meeting notes) and advised researchers to open the program space early and keep it open after the planned activities to make space for informal social interactions among program participants. In conceptualizing the healthy eating component, SGC members unanimously emphasized the importance of an engaging, interactive experiential format that uses food demonstrations and discusses food experiences/memories, rather than didactic education.

The SGC impacted the research plan for the RCT to test the program, through their input into discussions about outcome measures study recruitment, and data collection tools. During the eleventh SGC meeting, for example, the discussion focused on prioritizing trial outcomes and streamlining the interview guide, in response to testing the effectiveness outcome questionnaires with older adult SGC members. Their feedback was that the data collection interviews were too lengthy, and they had concerns about participant burden. In response, EMBOLDEN researchers asked SGC members which outcomes were most important to measure the program effectiveness and presented these findings to the group. Group discussion noted the importance of multiple outcomes, and the redundance of questions which were asked similarly across multiple measurement tools. SGC members asked whether it was possible to remove redundant questions, which led to a discussion about how changing tools can impact validity and reliability. Given this feedback, the researchers encouraged the team to focus on streamlining the number of outcome measures used rather than removing items within a tool; some outcome measures being considered were removed as a result. The process of choosing outcomes that matter was brought up in a focus group:

At least one meeting we talked about choosing outcomes that matter to older adults and I think the older adult members of the SGC had … you know sort of added to that particular aspect looking at … you know sort of recognizing that what’s important to the researcher, what the researcher thinks would be an important result might not be an important result to the participant – Older adult 3, focus group 2.

The SGC had a significant impact on the decision to transition EMBOLDEN to virtual implementation in the pilot neighborhood. Pre-pandemic, the EMBOLDEN program was designed to be delivered in-person in the community. Early in the pandemic the researchers were prepared to delay implementation, until pandemic restrictions eased to the point of accommodating this in-person programming. Given the impacts of the ongoing COVID-19 pandemic, meeting notes captured strong assertions from older adult SGC members:

[The] pandemic positions internet access as a human right… it is the responsibility of city planners [and] governments to bridge the gap for those who cannot communicate virtually during COVID-19, this is how governments prepare communities for the future... By not starting the program until after 2021, we exclude everyone vs. virtual delivery that excludes some people who are not willing/able to participate virtually – 10^th^ SGC meeting notes.

The SGC also advised EMBOLDEN researchers not to underestimate the time, effort and coaching required to support older adults to participate in virtual community programs. This suggestion led the research team to propose a technology lending library as part of a successful grant application. The research team created education materials and ensured that staff were available to coach those who needed it to help address this technology access barrier.

### Translational impacts

During the evaluation period, SGC members worked with researchers to provide input to a variety of knowledge translation products including infographics [54, 55], manuscripts [56, 57], training and program materials, research briefs [58, 59], and the study website. SGC members have co-authored and co-presented webinars and academic conference presentations with EMBOLDEN researchers.

### Impacts on participants

Evaluation participants spoke about the impact that research involvement had on their lives. Some participants expressed partnering in the EMBOLDEN study provided opportunities to learn and exchange knowledge, increased their interest in being involved as a research partner in future projects, and changed their health behaviors e.g., by increasing physical activity.

## Discussion

The aim of this study was to evaluate the engagement of older adults and health/social service providers which occurred as part of an ongoing, extensive effort (16 meetings over 21 months) to co-design a mobility intervention for older adults. Guided by DE, (which is known as a participatory approach to evaluation [33, 34]), older adults from the SGC set evaluation priorities, chose methods of data capture and tailored measurement tools for use in the engagement evaluation. This study responds to calls in the literature for clear reporting of the impact of patient-engaged approaches to research [4, 6–9]. Our findings offer insights on the structure of engagement efforts, the process of engaging and the impacts of engagement across stages of research.

### Structure

This project responds to calls in the literature for research partnerships inclusive of ethnically diverse partners [21, 60]. Diversity was identified as an important feature of the EMBOLDEN study, as exemplified by the suggestion from older adult SGC members to include it as an evaluation domain. This suggestion is aligned with previous research that found that diversity of culture, background, knowledge and skills which mirrors that of the larger population contributes to the longevity, and success of community-based research partnerships [61]. Across data sources, evaluation participants commonly mentioned the diversity of gender, ethnic, and disciplines among service providers as a strength of EMBOLDEN’s SGC. Similar to other patient partner populations, most evaluation questionnaire respondents were female and well-educated [62, 63], but our results showed some diversity of age, and ethnicity and with some representation from visible minority; lesbian, gay, bisexual, transgender; new immigrant; and disability communities. This diversity was more pronounced among older adult than service provider questionnaire participants. The diversity of SGC questionnaire respondents is notable considering the lack of age, ethnicity, and birth country diversity among patient partner populations in Canada and the UK [62, 63].

### Process

Identified strengths in the process of engagement within EMBOLDEN’s SGC included a welcoming, inclusive atmosphere that encouraged discussion, and well-organized meetings with clear communication that avoided jargon. Reports of engagement in EMBOLEN’s SGC were described as non-hierarchical, inclusive, and trusting which contrasted with other studies that reported more consultative engagement, with less power-sharing during the COVID-19 pandemic [64, 65]. Inclusivity and clear communication have been previously identified as enablers to patient engagement [66–68], and organized facilitation in meetings has been described as an enabling feature of the ‘management’ of engagement processes [69]. Recent work suggests that skillful meeting facilitation may be even more important when working virtually, and crucial for remote or virtual engagement activities [70, 71].

The rapid scale up of videoconferencing since 2020 has led to research on videoconference or ‘zoom fatigue’, defined as ‘*the somatic and cognitive exhaustion caused by intensive use of videoconferencing tools, frequently accompanied by related symptoms such as tiredness, worry, anxiety, burnout, discomfort, and stress, as well as other bodily symptoms such as headaches’ *[72]. Studies have shown that videoconference fatigue is not just a function of overall time spent in meetings [73], and propose that since the human brain naturally compensates for missing information during videoconferences, (which lack eye contact, body language and the true synchronization of in-person communication), more cognitive effort is spent compensating, creating fatigue [72]. A recent study of more than 200 patient and public research partners, and 65 engagement practitioners on remote patient and public involvement, found that screen fatigue may be felt more acutely by engagement practitioners than by the patient and public partners [70].

SGC members who participated in evaluation activities identified a double-edged sword of virtual engagement: the convenience of not having to leave one’s home to meet, though engaging with long meetings staring at screens is a tiring format. At least one evaluation participant in our study recognized the toll that screen fatigue took on their colleagues, and many mentioned screen fatigue as a limitation of virtual SGC meetings. Evaluation participants felt that virtual engagement was strengthened by the group’s familiarity with one another, facilitated by having three in-person meetings before transitioning to virtual engagement. We also heard that being automatically placed into smaller virtual break-out groups was convenient, allowed for more open conversation, which resulted in more engaging meetings and served as a venue for sharing (what participants perceived as) the SGC’s most useful advice to researchers 

Interestingly, there was limited discussion of trust, and few concerns brought up about power-sharing by evaluation participants. This is notable considering that a recent Canadian study noted power imbalances as the most commonly experienced barrier for patient and public research partners working in health systems [62], and the emphasis in the literature on trust and the effort required to address power differences between researchers and research partners [74, 75]. Previous research has shown that efforts to be inclusive, such as break-out groups, and an informal environment can reduce power differentials between researchers and research partners and increase trust [67, 76]. Given that these features were identified as strengths of EMBOLDEN’s approach to engagement, a trusting and power-neutral environment may have been created, potentially explaining the absence of these important attributes in the engagement evaluation results.

Another possible explanation for the limited discussion regarding trust and power differentials among evaluation participants could be related to the timing of engagement evaluation. The evaluation period included meetings that occurred between December 2019 and February 2022, including early in the COVID-19 pandemic, when many programs and services for older adults had paused or pivoted to virtual offerings. Our team encountered challenges conducting environmental scanning, as community organizations’ websites often reflected pre-pandemic schedules, making it difficult to discern which programs were available, and in what format. This scarcity of information may have had the effect of redistributing power between researchers and research partners. The service providers and older residents of EMBOLDEN’s SGC had unique expertise and insight into how the pandemic was affecting neighborhood-level service provision and, in turn, older adults. This information was invaluable to EMBOLDEN researchers and hard to access through other forms of public information at that time.

Table 1, row 2, mentions processes, referring to one of the earliest activities completed by SGC members- co-developing the Terms of Reference for the SGC (TOR). The draft TOR were a summary of a 20-min discussion (with 5 breakout groups) during the first gathering of potential SGC members and researchers about their values, interests, goals and expectations of working together. Revisiting the TOR provides insight into how EMBOLDEN’s SGC processes may have influenced the creation of a trusting environment. The TOR outlined four features of what working together will look like: successful partnerships (based on listening, respect and valuing each other’s experiences), collaborative decision-making (that shares how decisions are made, and avoids top-down choices), clear communication (with frequent updates), and understanding (each other, the commitment, and logistics of EMBOLDEN). These co-developed features meant that transparency in decision-making, acknowledging contributions, timely sharing of meeting notes, valuing all voices, thanking members contributions, and offering honorariums to recognize lived expertise were planned aspects of SGC processes that could explain how EMBOLDEN’s engagement was perceived as a strength. Table 1’s counts of attendance reflect how many SGC members and researchers were present at each meeting. Except for the first meeting, SGC members made up between 67 and 80% of meeting attendees. The conscious effort to ensure that SGC members always made up most of the attendees at meetings (by only involving research team members if their expertise corresponded with meeting topics vs. every research team member attending every meeting) may have also contributed to the trusting dynamic and atmosphere conducive to discussion identified in the evaluation.

### Impacts

Older adults from the SGC felt it was important for the evaluation to measure the impact of the SGC across different stages of the research process. This view corresponds with recent research which found that patient partners’ perception of their impact was the most influential feature in determining whether they had a positive experience in research [67]. Previous authors have outlined how important it is for researchers to communicate with partners about how their contributions are used, valued and ultimately impactful [67, 77]. This participatory evaluation may have served as a mechanism to track, reinforce, and value research partners contributions, and may have contributed to the endurance of the partnerships within SGC, as evidenced by the consistent attendance of members over the two-year evaluation period.

Previous studies have categorized patient partner impacts across different stages of research, finding that it may be more common for partners to be engaged in select research activities (such as choosing outcomes or advising on recruitment strategies) as opposed to continuous engagement throughout all stages of research [78, 79]. EMBOLDEN’s SGC may be unique in this sense, as evaluation results demonstrate that SGC partners impacted all stages of the EMBOLDEN study. Advice from older adults and service providers led to a more diverse, representative guiding council which impacted the preparatory phase of research by enhancing researchers understanding of target neighborhoods and ensuring the study used inclusive language that would resonate with older adults living in these areas.

The SGC interpreted research evidence, impacting how program features should be operationalized; iteratively refined the outcome measurement plan; and shaped recruitment processes during the execution phase of research. These findings correspond with previous studies that have described the impact of research partnerships on tool development [27, 80, 81]; recruitment [82], and outcome measurement [83].

SGC members were impacted by their involvement in this co-design process and found similar results to [21, 84, 85] who describe a desire to continue or foster new research partnerships because of engagement in research. Research partners described how being involved with EMBOLDEN’s SGC impacted what they knew about physical activity, and how often they were active, which is aligned with previous findings about the effect of research partnership in promoting patients’ health [67, 86].

### Limitations

This study was limited by its approach to planning and evaluating engagement at a single (as opposed to multiple) time point(s). We partially addressed this limitation through our analysis of the notes from each of the SGC’s 16 meetings throughout the evaluation period which provided helpful insights into engagement processes over time. The impact of the SGC on the knowledge translation stage may be underreported, relative to other sections, as a variety of SGC contributions continued beyond the evaluation period of this study. Perspectives of service providers may be underrepresented, as their rate of participation in evaluation activities was lower than older adult SGC members. While the EMBOLDEN study maintained relational continuity and support with partner organizations, evaluation activities occurred at a time when the SGC saw turnover of around 7 service provider members, due to retirements and parental leave. Newer representatives may not have felt comfortable evaluating a project they just joined. We also heard from service providers during SGC meetings that the demand for their services was extraordinary during the COVID-19 pandemic, which meant they may have had less time for additional EMBOLDEN-related research activities, such as this sub-study, beyond their attendance at SGC meetings, which was consistent (see Table 1).

This study is limited by the lack of sociodemographic data collected on the diversity of the following groups: the older adults who helped design this evaluation, focus group participants and SGC members. This limits us from knowing how many different SGC members participated in different parts of the study. We encourage future reporting of diversity to distinguish between the sociodemographic characteristics of research partners, and those of research participants. This clarity will be especially important to ensure that research partnerships are as diverse and representative as the populations that will be most impacted by research results [60]. Additionally, the lead author was known to SGC members through her facilitation role at SGC meetings. Analysis was checked by a second researcher who did not attend SGC meetings and confirmed by all co-authors. It is common within DE for the lead evaluator and program stakeholders to be involved to design and undertake evaluation activities which are seen as internal team functions that occur iteratively [35].

### Implications

Though involving citizens and patients in health research is becoming more common, a 2018 review of more than 350,000 trials, showed that > 1% reported engaging patients in their research [87]. Other research reported that pragmatic trials, those based in the UK and trials involving children are more likely to have had patient involvement than others [88]. Results from the EMBOLDEN study, and this sub-study evaluating the codesign of the intervention will be of interest to trial researchers considering involving patients in their work.

Equity-seeking groups of older adults may have been traditionally excluded from patient engaged or partnered roles in research [21], and so the EMBOLDEN project’s efforts to engage older people from Hamilton neighborhoods with high health inequity (including low-income older people, isolated older people, new immigrant older adults) is an important contribution to the field of impact evaluation in patient and public engaged processes.

Developmental evaluation served as an accommodating methodological approach to measuring complex, extensive engagement, which may be characteristic of experience-based efforts to co-design programs and services. Using convergent mixed methods within DE allowed for a clear summary of results using a joint display. The utility of DE approaches to evaluate codesigned projects may be relevant given the National Health Service (NHS) recent guidance in the UK endorsing codesign as an approach among integrated care boards, trusts and policy [89].

## Conclusion

Older adults can make important contributions to improve health interventions, community programming and evaluation activities through their lived experience and connections to community. Impact evaluation of engagement activities is just as important to older adults as it is to researchers, funders, and policymakers. In partnership with a group of older adults, an evaluation was designed to determine the strengths, limitations and impacts of engagement during an extensive process to co-design a mobility intervention. This study contributes to a growing body of engagement impact evaluation research, integrating multiple data sources to identify strengths and limitations and trace impacts of research partners across stages of research and over time.

### Supplementary Information

**Additional file 1.** Supplementary table.

## Data Availability

The datasets used and/or analysed during the current study are available from the corresponding author on reasonable request.

## Appendix A: Designing the evaluation: discussion questions

- What is unique about EMBOLDEN’s Strategic Guiding Council that you think is important to capture in an evaluation?
- What challenges did you encounter to engaging in EMBOLDEN’s Strategic Guiding Council?
- What part of your engagement in EMBOLDEN’s Strategic Guiding Council went well?
- What changed for you as a result of you being a research partner on EMBOLDEN’s SGC?
- In what ways do you feel your engagement in the SGC influenced the EMBOLDEN program?
- In what ways do you feel your engagement in the SGC influenced EMBOLDEN researchers
- Some data sources we could use are: survey, interviews, focus groups, meeting notes, observation, metrics (#s of participants, time spent, # of meetings)- which do you see as most relevant to this project and why? Can you think of other any sources of data should inform the evaluation of the Strategic Guiding Council?
- What is most important for other researchers to know about engaging older citizens and service providers in research projects?
- How would you like to be involved in this work going forward?

## Appendix C: Focus group evaluation prompts

1. Think back to when you first joined EMBOLDEN’s Strategic Guiding Council, how prepared did you feel to contribute to this research project? What training, support, or past experiences helped you feel prepared? What additional training or support would you like to have received?
2. What is the main reason you want to contribute to this research project?
3. Patients and stakeholders can contribute to research projects in many ways. This could include:
  - Making sure researchers know what kind of information is important to older adults
  - Deciding what the study should be about;
  - Deciding who to include in the study;
  - Choosing what outcomes the study will measure;
  - Tracking study progress;
  - Sharing study findings

We call contributing to research projects in ways like this “being a research partner”. Were you a research partner on this project ? Describe.

4. Has your involvement in the project changed your life in any way? This might include things like building new relationships, better managing your health, or finding new work opportunities. If so, please share.
5. Sometimes there are challenges when researchers, older people, and other stakeholders work together. These might include finding a convenient time to meet or communicating clearly with each other. What have been the biggest challenges for you on this research project? What would you like to see improved?
6. What do you feel were the strengths of EMBOLDEN’s approach to engaging the Strategic Guiding Council?
7. Did you feel the EMBOLDEN project was well-organized? Why or why not? Did your perception of EMBOLDEN’s organization change over the course of your engagement in the Strategic Guiding Council?
8. Please share anything that helped you contribute to this research project. For example, this may include things you did to ensure your view was heard or things the researchers did to ensure everyone was included.
9. Based on your experience with this research project, what would you suggest be done to help others contribute as research partners?
10. Prior to working on this project, had you contributed to a research study as a research partner?
11. Have you worked with any of these researchers before this project? If yes, for how long have you worked with these researchers?
12. If the opportunity arose, would you be interested in working as a research partner on another research study? Why or why not?
13. COVID-19 restrictions meant the Strategic Guiding Council had to begin meeting online as opposed to in-person. Did you have the support you needed to engage online? What are the strengths of a virtual approach? What are the weaknesses of this approach? What could have been done differently?

## Abbreviations

SPOR: Strategy for Patient Oriented Research
CBPR: Community-based participatory research
CIHR: Canadian Institutes for Health Research
EBCD: Experience-based co-design
EMBOLDEN: Enhancing Physical and Community MoBility in OLDEr Adults with Health Inequities Using CommuNity Co-Design
WE-ENACT: Ways of Engaging- Engagement Activity Tool
DE: Developmental Evaluation
LGBTQ: Lesbian, gay, bisexual, transgender, queer
GRIPP2: Guidance for reporting involvement of patients and the public
OA: Older adult
PCORI: Patient-Centered Outcomes Research Institute
PEIRS: Patient engagement in research scale
PPEET: Patient and public engagement evaluation tool
RCT: Randomized controlled trial
REB: Research ethics board
SP: Service provider
SGC: Strategic Guiding Council
TOR: Terms of reference
